# Glass Fiber-Reinforced Phenol Formaldehyde Resin-Based Electrical Insulating Composites Fabricated by Selective Laser Sintering

**DOI:** 10.3390/polym11010135

**Published:** 2019-01-14

**Authors:** Zhaoqing Li, Wangbing Zhou, Lei Yang, Peng Chen, Chunze Yan, Chao Cai, Hua Li, Lee Li, Yusheng Shi

**Affiliations:** 1 State Key Laboratory of Materials Processing and Die & Mould Technology, School of Materials Science and Engineering, Huazhong University of Science and Technology, Wuhan 430074, China; aimouse@msn.com (Z.L.); 18756091538@163.com (W.Z.); jackyejackye@163.com (L.Y.); peng_chen@hust.edu.cn (P.C.); shiyusheng@hust.edu.cn (Y.S.); 2 State Key Laboratory of Advanced Electromagnetic Engineering and Technology, Huazhong University of Science and Technology, Wuhan 430074, China; leehua@mail.hust.edu.cn (H.L.); leeli@hust.edu.cn (L.L.)

**Keywords:** additive manufacturing, selective laser sintering, surface modification, ternary composites, mechanical properties, electrical properties

## Abstract

In this study, glass fiber (GF)/phenol formaldehyde resin (PF)/epoxy resin (EP) three-phase electrical insulating composites were fabricated by selective laser sintering (SLS) additive manufacturing technology and subsequent infiltration. In the three-phase composites, glass fibers modified by a silane coupling agent (KH-550) were used as reinforcements, phenol formaldehyde resin acted as the binder and matrix, and infiltrated epoxy resin was the filler. Mechanical and electrical properties such as tensile strength, bending strength, dielectric constant, electrical conductivity, and electric breakdown strength of the GF/PF/EP three-phase composite parts were investigated. The results indicated that after being infiltrated with EP, the bending strength and tensile strength of the GF/PF/EP composites increased by 30% and 42.8%, respectively. Moreover, the flexural strength and tensile strength of the GF/PF/EP composite increased with the increase of the glass fiber content. More importantly, the three-phase composites showed high electrical properties. Significant improvement in the dielectric constant, electric breakdown strength, and resistivity with the increase in the content of glass fiber was observed. This enables the prepared GF/PF/EP composites to form complex structural electrical insulation devices by SLS, which expands the materials and applications of additive manufacturing technology.

## 1. Introduction

As an important thermoplastic engineering material, phenol formaldehyde resins (PF) are usually used in combination with organic or inorganic fibers and fillers [1]. The curing of these combined materials provides excellent electrical insulation, thermal stability, flame retardancy, and heat resistance properties [2,3,4,5]. Glass fiber (GF)-reinforced phenol formaldehyde resin-based composites have been widely applied in radar radome, automobile fittings, printed circuit board, and so on by virtue of their light weight, high specific strength, and excellent electrical and thermal insulation properties [6,7,8]. The reinforcing effect of glass in phenol formaldehyde resin has been evaluated at various glass fiber loadings. Tensile strength, tensile modulus, and flexural strength increases with an increase in fiber loading [9]. Cui et al. prepared high-performance GF/PF composites with a bio-oil addition of 20% based on a hand lay-up process [8]. The rapid growth of PF-based composite applications has inspired extensive research to improve the fabrication and performance of such composites.

For the fabrication of glass fiber-reinforced resin-based composites, such conventional methods as hand lay-up, molding, and extrusion molding have the advantages of high production efficiency and high product precision and also the disadvantages of requiring mold design, long preparation cycles, and high production cost [10]. In addition, it is tricky to manufacture parts of complex geometric shapes with traditional methods due to the complex surfaces and internal structures.

With selective laser sintering (SLS), a powder bed fusion additive manufacturing (AM) process [11], three-dimensional (3D) objects can be fabricated by adding powdered materials layer by layer according to computer-aided design (CAD) models. The main advantage of SLS is that it can process an extensive scope of materials including polymers, metals, ceramics, and composites [12,13,14]. In these SLS materials, polymers are most widely used and also show a broad application prospect, due to their diversity in species, performance, and application of various modification technologies [15]. With the development of SLS technology, there is a growing need to develop and be able to process a much greater variety of materials than is currently possible. The handful of current polymeric materials that exist for processing by SLS techniques does not meet the requirements of the majority of commercial products [16]. Recent research in SLS processing of polymers includes polyamides (PA12 and PA11) [17,18,19], polypropylene [20], and PEEK [21]. Many studies have focused on optimizing the process parameters, as well as studying the effects of SLS process parameters on the mechanical properties of components made from neat polymers [15,22]. However, the widely acknowledged stumbling block in the progression of SLS technology for future applications is the limited range of materials, especially for thermal and electrical applications polymers. None of the reported studies have focused on electrical insulation fillers with an aim of enhancing the electrical insulation of the resulting composite. Therefore, it is of significance to study the forming and electrical properties of glass fiber-reinforced phenol formaldehyde resin-based electrical insulating composites fabricated by SLS.

In this work, SLS technology was employed to fabricate GF/PF/epoxy resin (EP) three-phase composite materials. First, phenol formaldehyde resin was completely dissolved in organic solvents, and then, glass fiber was added to prepare the raw materials. Resin-coated fiber powder suitable for laser selective sintering was prepared through ball milling, drying, crushing, and sieving. After the SLS process, a green part having a certain porosity and mechanical strength was fabricated. The final part was then obtained by impregnating the epoxy resin and curing. Mechanical and electrical properties of the GF/PF/EP composites were investigated in this paper. This study lays the foundation for rapid fabrication of various GF/PF/EP composite electrical insulation parts of different shapes and sizes.

## 2. Experimental

### 2.1. Materials

Alkali-free glass fiber (Class E, 300 mesh) was supplied by Fuhong Mineral Products Co., Ltd. (Beijing, China). Phenol formaldehyde resin (PF-2123) was obtained from Mingyang Bonding Material Co., Ltd. (Wuxi, China). Model PF-2123 was a kind of yellow thermosetting phenol formaldehyde resin powder mixed with an appropriate amount of urotropine. The characteristics of the PF-2123 phenol formaldehyde resin are shown in Table 1. Curing agent methyl tetrahydrophthalic anhydride (Me-THPA) and curing accelerator (DMP-30) were provided by Sanmu Group (Jiangsu, China). Other inorganic chemicals used in this study, such as silane coupling agent (KH-550) and ethanol, were reagent grade and used as obtained without any purification.

### 2.2. Experimental Process

**Figure 1:**
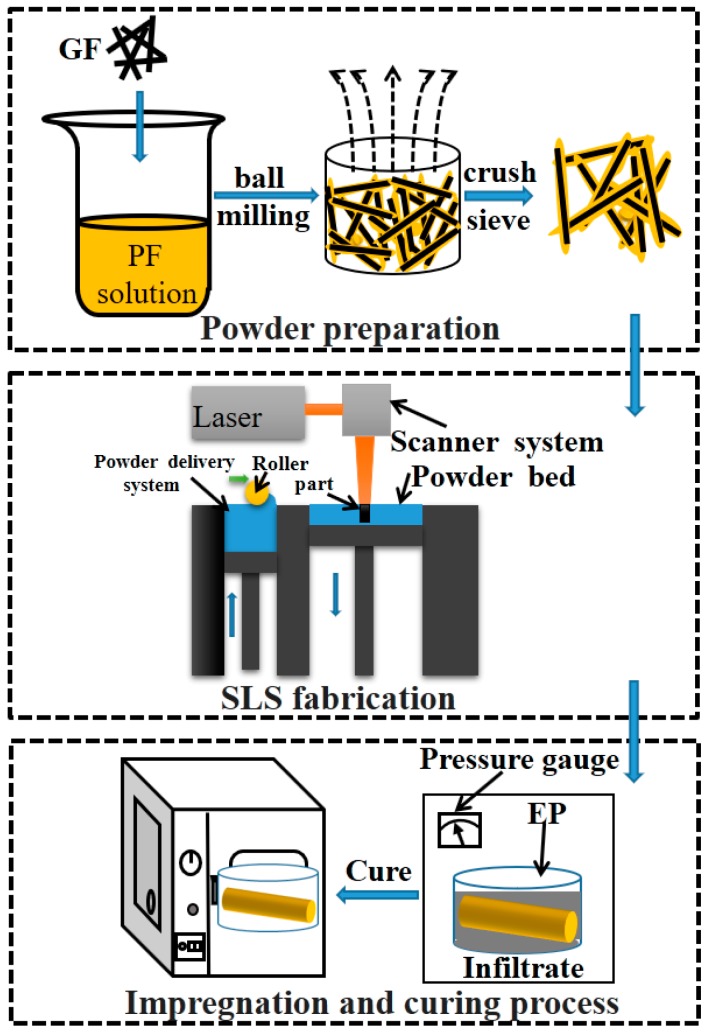
Procedures for the fabrication of the glass fiber (GF)/phenol formaldehyde resin (PF)/epoxy resin (EP) three-phase composite components.

Figure 1 shows the procedures of the process for fabricating the components of the GF/PF/EP three-phase composite. The experimental process mainly consisted of three steps, powder preparation, SLS fabrication, and the impregnation and curing process.

**Figure 2:**
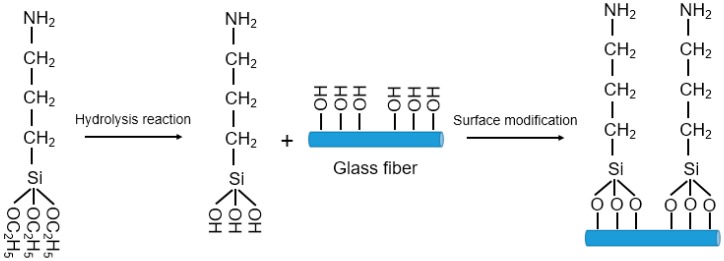
Schematic diagram of the silane coupling agent-modified glass fiber surface.

**Figure 3:**
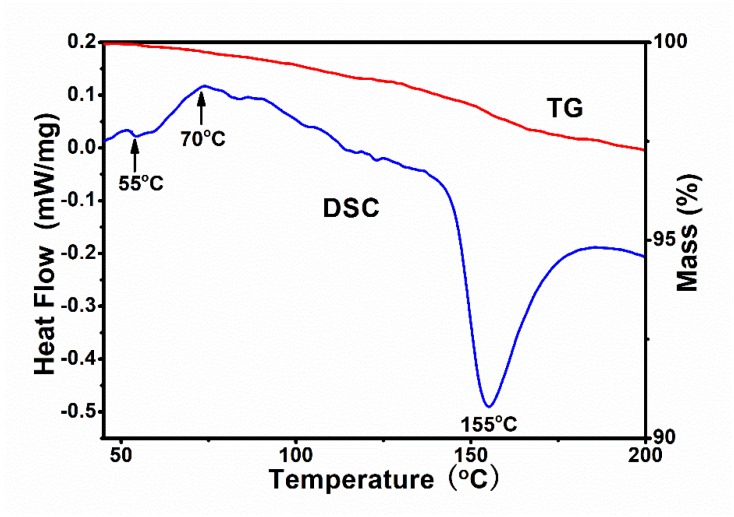
Thermogravimetric-differential scanning calorimetry (TG-DSC) curves of phenol formaldehyde resin.

(1) Surface treatment of glass fiber: Glass fiber tends to adsorb water vapor, dust, and other substances due to its low free energy and the less oxygen-containing polar group on its surface. This greatly reduces the bonding effect between the two phases of the glass fiber and the resin, which, as a result, significantly reduces the mechanical strength and electrical properties of the composite material [23]. In this work, the glass fiber was surface-modified with a silane coupling agent KH-550 to improve the interfacial bonding strength between the fiber and the resin [24]. The surface treatment experiments were as follows: (i) The glass fibers were placed in a muffle furnace and heated to 300 °C for 1 h. After that, they were ultrasonically washed 3 times with deionized water and absolute ethanol, respectively. Finally, the glass fibers were dried at 80 °C for 6 h. (ii) 95 wt.% absolute ethanol and 5 wt.% deionized water solution were prepared, and an appropriate amount of silane coupling agent was then added until the concentration of the silane coupling agent in the mixed solution reached 2 wt.%. (iii) Finally, the glass fibers were immersed in the mixed solution for 6 h, and the excess silane coupling agent on the surface of the glass fibers was removed with deionized water. The glass fibers were surface-modified after being dried in an oven at 110 °C [25]. The mechanism of modifying the glass fiber’s surface with the silane coupling agent is illustrated in Figure 2.

(2) SLS powder preparation: The phenol formaldehyde resin was dissolved in absolute ethanol. After it dissolved completely, the glass fibers were added. The mixture of phenol formaldehyde resin and glass fibers was put in a ball mill machine at 300 r/min for 1 h and then dried at 50 °C for 24 h. Subsequently, the dried mixture was pulverized and sieved to obtain a powder material suitable for SLS. In this work, GF/PF composite powders having a glass fiber volume content of 60 vol.%, 70 vol.%, and 80 vol.% were prepared, respectively.

(3) SLS process: As shown in Figure 3 of the thermogravimetric-differential scanning calorimetry (TG-DSC) curve of phenol formaldehyde resin, the phenol formaldehyde resin has a glass transition temperature *T*_g_ of 55 °C and a viscous flow temperature *T*_f_ of 70 °C. In the process of SLS, the preheating temperature selected for the powder should be between *T*_g_ and *T*_f_ [26].

The SLS experiments of GF/PF composites powder were conducted with a HK S320 SLS system equipped with a continuous wave 30W CO_2_ laser (the system is developed by Huazhong University of Science and Technology, Wuhan, China). Warpage is common in the SLS process, and researchers have tried different methods to minimize the warping effect [27]. The scan algorithm of the HK S320 SLS system is fixed. Therefore, we use optimized process parameters to solve the warpage problem. First, we determine the bed temperature via an experiment. When the bed temperature is lower than 65 °C, the formed parts are prone to warpage. When the bed temperature is higher than 65 °C, the surface of the part more easily adheres to the powder and causes low precision. Thus, we used 65 °C as the bed temperature. Other experimental results are shown in Table 2.

**Figure 4:**
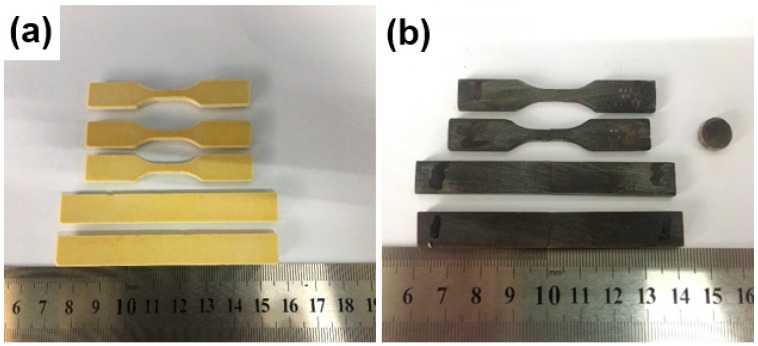
SLS parts before impregnating the epoxy resin (**a**) and after the impregnating and solidification (**b**).

Based on the series of preliminary experiments, the SLS process parameters were optimized and set as follows: bed temperature, 65 °C; laser power, 14 W; scanning speed, 3500 mm/s; scanning space, 0.3 mm; and layer thickness, 0.1 mm. The parts formed with optimized parameters are shown in Figure 4.

(4) Infiltration experiment: After the green part was fabricated with the SLS process, the thermoplastic phenol formaldehyde resin reacted with methenamine for curing at 180 °C for 2 h and then was impregnated with epoxy resin. The infiltration process was as follows: the epoxy resin and the curing agent Me-THPA were heated to 100 °C separately and then mixed. After that, the quantitative accelerator DMP-30 was added dropwise. The mixture was quickly stirred and poured into the container in which the green part was placed, and the liquid surface did not pass over the upper surface of the green part. Vacuuming was performed in a vacuum oven at 100 °C to fully infiltrate the pores of the sample. After 30 min, the sample was taken out. After removing the excess liquid resin on the surface, the sample was placed in an oven for curing. After cooling, the final part was obtained.

### 2.3. Characterization

The powder morphology and cross-section characteristic of the SLS specimens were observed using Sirion 200 and Quanta 200 scanning electron microscopes (Netherlands FEI Instrument, Eindhoven, Holland). These specimens were sputter coated with platinum under vacuum conditions for 300s to avoid charging. Thermal analysis experiments were conducted under a nitrogen protecting atmosphere using a Diamond DSC (PerkinElmer Instruments, Norwalk, CT, USA) to determine various melting and crystallization properties at a heating and cooling rate of 10 °C/min. To identify the nature of the bondings, the Fourier transform infrared spectra (FTIR) of the glass fiber before and after the surface modification were measured with a Bruker VERTEX 70 Fourier transform infrared spectroscope (Bruker, Billerica, MA, USA) and collected in the frequency range of 400–4000 cm^−1^. The porosity of the GF/PF green parts was measured by the drainage method [28]. First, the weight of the green parts was taken (*W*:g). Then, the green parts were degassed in distilled water under a constant vacuum pressure at room temperature (25 °C) for 24 h. After that, the samples were removed from the vacuum chamber, the water on the outer surface was removed, and the weight after adsorption was obtained (*W_a_*:g). The sample was immersed in distilled water to obtain the submerged weight of the samples (*W_s_*:g). The porosity of the sample can be calculated by the following formula:

$$P=(W_{a}-W)/(W_{a}-W_{s}).$$

For the determination of the mechanical properties, standard ASTM D638-03 Type V was utilized. The tensile and bending tests were performed on a Zwick/Roll Z020 instrument (Zwick, Ulm, Germany). The dielectric constant and dielectric loss of the GF/PF/EP green parts with a three-dimensional size of 40 mm × 40 mm × 1 mm were measured with a Concept40 broadband dielectric resistance spectrometer manufactured by Novocontrol, Montabaur, Germany. The KEITHLEY 2290E-5 high voltage power supply was used as a voltage applying device, and the KEITHLEY 6517B electrometer was a current measuring device for measuring the conductivity of the GF/PF/EP green parts as a function of frequency. The breakdown voltage of the GF/PF/EP green parts with a three-dimensional size of 80 mm × 80 mm × 1 mm was measured with a MS2673-IIB insulating oil dielectric strength automatic tester produced by Nanjing Minsheng Electronic Instrument Co., Ltd (Nanjing, China). During the test, the sample was tightly held between the two planar copper electrodes in the oil cup and immersed in the insulating oil to prevent the surface breakdown of the sample. The breakdown voltage value was divided by the sample size to obtain the volume resistivity.

## 3. Results and Discussion

### 3.1. Powder Characteristics

**Figure 5:**
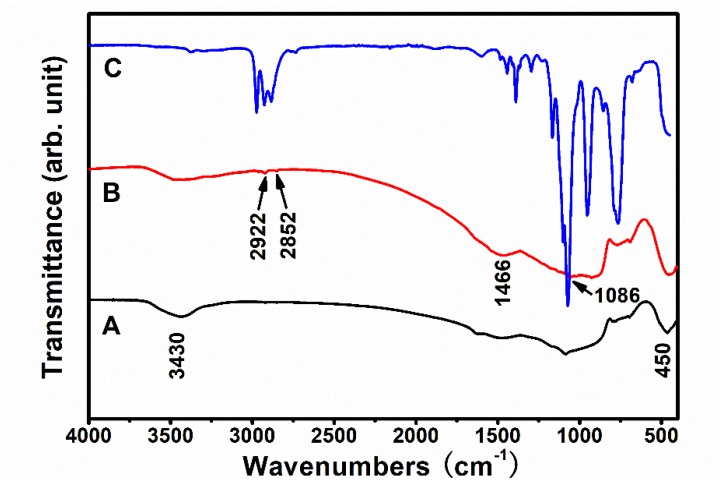
FTIR spectra of the glass fiber (A) before and (B) after the modification and (C) the silane coupling agent KH-550.

The FTIR spectra of the glass fiber before and after modification and the silane coupling agent KH-550 are plotted in Figure 5. In all the FTIR spectra, the signals between 400 and 800 cm^−1^ are from the deformation vibration of Si–O; the broad absorption band in the 3000–3600 cm^−1^ range should be assigned to the deformation and O–H stretching vibrations of the weak-bound water [29]. It can be concluded that the glass fiber before and after modification and KH-550 all contained Si–O bonds, and the presence of O–H bonds can be attributed to the physically adsorbed water. The water in the samples would be physically adsorbed from the air before the FTIR measurements after calcination. Compared with curve a in the infrared spectrum, curve b has peaks of 1086, 1466, 2852, and 2922 cm^−1^ more than the curve a, corresponding to the stretching vibrations of the Si–O–Si bond, the deformation vibrations of the –NH_2_, and the symmetric and anti-symmetric stretching of the –CH_2_–, respectively [8,30]. Therefore, the presence of these groups on the surface of the modified glass fiber indicates that KH-550 was successfully coupled to the surface of the glass fiber [31].

**Figure 6:**
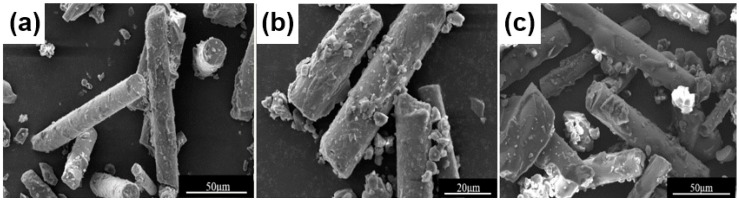
SEM micrographs of phenol formaldehyde resin-coated glass fibers with glass fiber contents of (**a**) 80%, (**b**) 70%, and (**c**) 60%.

A uniform coating of phenol formaldehyde resin on the surface of glass fiber can significantly increase the contact area between the reinforcing phase and the matrix resin [32]. When the two-phase dispersion uniformity of glass fiber and phenol formaldehyde resin is poor, the aggregation of the glass fibers causes the composite material to be segregated, in which case the mechanical properties of the composite material would be reduced. Therefore, the dispersion state of the composite powder becomes an important indicator in evaluating the quality of the powder. The SEM image in Figure 6 shows the morphology of phenol formaldehyde resin-coated glass fibers in three different ratios. It can be found that the phenol formaldehyde resin is uniformly coated on the surface of the glass fiber, which will facilitate the subsequent SLS process.

In the process of fabricating the green parts through SLS, the low bulk density of the powder on the powder bed may result in certain porosity of the sintered part, which makes the properties of the green parts descend [33]. B.V. Hooreweder et al. [34] compared the structure and properties of SLS- and injection-molded parts using the same material. By observing the cross section of the parts, it was found that there were more pores in the SLS parts, which also showed poor mechanical properties. In this work, the porosity of the GF/PF green parts measured by the drainage method for different fiber contents is listed in Table 3. It can be seen that the addition of fiber can increase the porosity of the GF/PF sintered sample. This is because the degree of compaction between the fibers is lower than that of the pure resin, and the higher the fiber content, the lower the degree of contact between the fibers. Therefore, the sintering of the powder forms a sintered sample having a high porosity. Moreover, the apparent viscosity of the GF/PF composite powder at the glass transition temperature *T*_g_ is large, so it cannot be completely sintered at the time of rapid laser sintering, which results in a low density and low strength of the green parts. In this paper, the density of the sample was enhanced by impregnating the epoxy resin to obtain the GF/PF/EP three-phase material, which is beneficial to improve the mechanical and electrical properties of the part.

**Figure 7:**
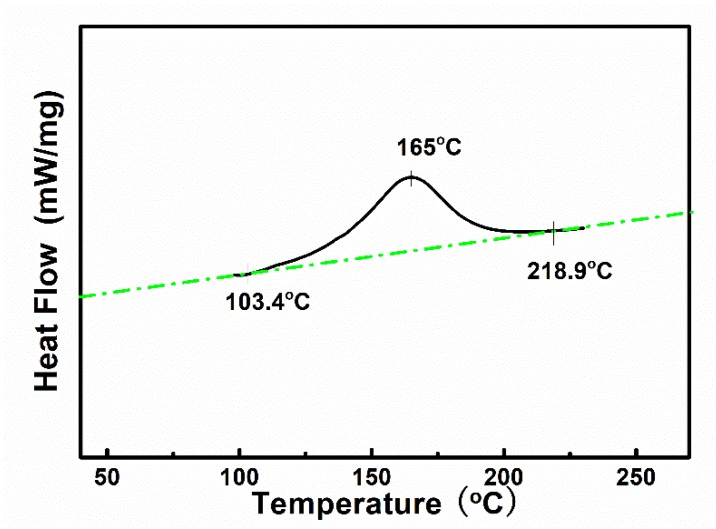
DSC curve of epoxy resin E51.

Figure 7 shows the DSC curve of the epoxy resin E51. According to the curve, the curing peak of the epoxy resin is in the range of 103–218 °C, and the top temperature of the peak is 165 °C. In order to fully cure the epoxy resin, a curing method with a temperature gradient was adopted. The curing step is as follows: holding the sample at 120 °C for 5 h, at 150 °C for 3 h, and at 200 °C for 2 h. The ratio of each component of the impregnated resin is E51/Me-THPA/DMP-30 = 100:75:0.15.

### 3.2. Mechanical Properties

Table 4 shows the mechanical properties of the GF/PF/EP three-phase composites with different fiber contents fabricated through SLS. The GF/PF/EP samples with varied glass fiber volume fractions (60 vol.%, 70 vol.%, and 80 vol.%) showed tensile strengths of 86.4, 92, and 96.2 MPa and bending strengths of 119, 129, and 137 MPa, respectively.

It can be concluded that the mechanical properties of the GF/PF/EP composites are enhanced with the increase of the GF content. For the fiber-reinforced resin composite, the crack generated by stress will expand, but the matrix resin can transfer the stress to the high-strength fiber through the interface [7,35]. The adhesion of the resin matrix to the glass fibers hinders the extraction and the bridging of the fibers and so on, all of which inhibit the crack propagation [36,37]. With higher fiber content in composite material, under the action of stress, the expansion of the fiber hinders the crack, so larger external forces will be needed for the fiber pull-out and de-bonding. In this way, the mechanical strength, such as the bending and stretching of the composite, is significantly improved.

**Figure 8:**
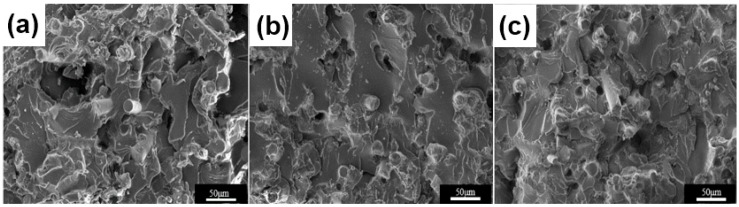
SEM micrographs of the cross section of composite material with a glass fiber content of (**a**) 80 vol.%, (**b**) 70 vol.%, and (**c**) 60 vol.%.

SEM photographs of the GF/PF/EP samples with varied glass fiber volume fractions of 80 vol.%, 70 vol.%, and 60 vol.% are presented in Figure 8. The fracture of the pure epoxy resin is brittle and a smooth fracture. However, the section of the GF/PF/EP sample is uneven, and the surface of the substrate has a cloud-like shape, which indicates that the toughness of the material is improved, and the fiber has a toughening and reinforcing effect. By comparing the cross-sectional views of the sintered samples, it can be found that the fibers have good dispersibility in the matrix, the GF/PF/EP is closely bonded, and there is almost no gap. There is a small number of holes in the section due to the fiber being pulled out. Because the volume content of the fibers is not much different, the morphology of the GF/PF/EP section does not change much.

### 3.3. Electrical Properties

Dielectric property, one of the most important properties of glass fiber-reinforced composites [38], is a polarization phenomenon caused by free charge in the material and the influence of the electric field on the dipole (the negative charge moves toward the positive electrode, and the positive charge moves toward the negative electrode). Therefore, the dielectric properties of the composite are closely related to the frequency and temperature, as well as the dielectric properties and volume fraction of each component [39].

**Figure 9:**
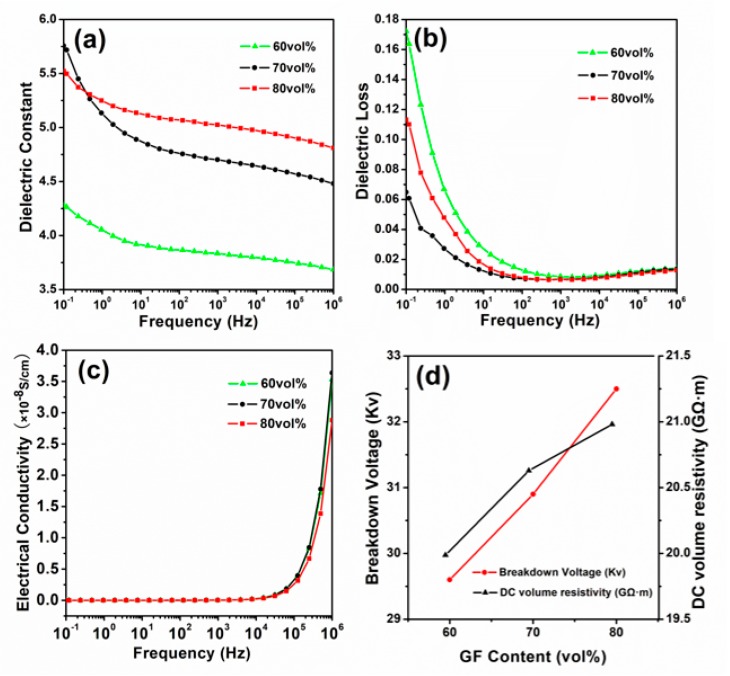
The (**a**) dielectric constant, (**b**) dielectric loss, (**c**) electrical conductivity, and (**d**) breakdown voltage and DC volume resistivity of the composites changed with fiber content and frequency at room temperature.

Figure 9 shows the dielectric properties of the glass fiber-reinforced materials with different fiber contents from 0.1 Hz to 1 MHz. Among them, Figure 9a–c show the dielectric constant, dielectric loss, and electrical conductivity as a function of frequency, and Figure 9d shows the trend of DC volume resistivity and breakdown voltage versus glass fiber content. The irregularly distributed charges and dipoles inside the dielectric are subjected to an external electric field to rearrange the orientation (i.e., orientation polarization), and the bound charges appearing on the surface of the medium cause the entire medium to exhibit a macroscopic dipole to generate a polarized electric field. Polarization is divided into two categories, one is the displacement polarization of the electrons and ions, which occurs at a frequency of 10^9^ to 10^16^ Hz. The other type is dipole orientation polarization and interfacial polarization, which occurs at a frequency of 10^−3^–10^8^ Hz. Displacement polarization is instantaneous, while orientation polarization and interfacial polarization are processes with a certain time [40].

When the frequency is low, there is enough time for polarization, and the dielectric constant is a large value [41]. At higher frequencies, the orientation polarization is delayed by the inertia to the electric field, and the dielectric constant is gradually reduced, resulting in a decrease in the dielectric constant of the dielectric constant of Figure 9a with increasing frequency. The dielectric constant of the sample with a fiber content of 60 vol.% decreased from 4.28 to 3.68, from 5.76 to 4.48 at 70 vol.% and from 5.52 to 4.81 at 80 vol.% a frequency of 0.1 Hz to 1 MHz. The increase in content increased, which may have been due to an increase in the interfacial polarization [42].

As shown in Figure 9b, at the frequency of 0.1 Hz, the dielectric loss of the sample was 0.17 at a fiber content of 60 vol.%, 0.065 at 70 vol.%, and 0.11 at 80 vol.%. The dielectric loss of the samples with three different fiber contents decreased sharply with the increase of frequency and then remained basically unchanged. Dielectric loss is composed of conductance loss, polarization loss, ionization loss, and structural non-uniform loss. The dielectric loss drops sharply before 1 KHz. This is mainly dominated by the conductance loss, which is the loss caused by the leakage current through the medium under the action of an electric field. Between 1 kHz and 1 MHz, there is a slight increase in dielectric loss. This process is the energy loss caused by the composite during polarization, because there is a loss peak in the polarization loss.

The composite materials with different fiber contents in Figure 9c had the same electrical conductivity, indicating that a small change in the fiber content has little impact on electrical conductivity, and the conductivity of the composite materials with the three fiber contents had the same trend. The conductivity consisted of direct current conductivity and polarization conductivity. The conductivity of the sample was 2.5 × 10^−13^ at 0.1 Hz and 6.0 × 10^−10^ at 1.0 × 10^4^ Hz. Conductivity at 1 MHz was 3.5 × 10^−8^. At lower frequencies, the conductivity remained essentially the same, which was dominated by the DC conductivity provided by the carriers. When the frequency was increased to a higher level, the conductivity was significantly increased, and the polarization conductivity formed by the polarization of the bound charge was dominant.

According to Figure 9d, for the composite materials having a fiber content of 60 vol.%, 70 vol.%, and 80 vol.%, the breakdown voltage was 29.6, 30.9, and 32.5 KV, respectively, and the DC volume resistivity was 19.978, 20.629, and 20.981 GΩ·m, respectively. This reveals that the breakdown voltage and DC volume resistivity of the composite increased with the increase of the fiber content. This is because the main chemical component of glass fiber is silica, which has a high electrical resistivity and is a good insulator. At the same time, as the fiber content increases, the effect of interfacial polarization enhances, resulting in an increase in the dielectric constant and a harder breakdown of the dielectric. Therefore, an increase in the GF fiber content is advantageous for improving the electrical insulation properties of the composite material.

## 4. Conclusions

GF/PF/EP three-phase composites were fabricated through SLS and subsequent infiltration. After investigating the mechanical and electrical properties, such as tensile strength, bending strength, dielectric constant, electrical conductivity, and electric breakdown strength of the GF/PF/EP composites, the following was found:

- The laser selective sintering fiber-reinforced resin composite material has a certain porosity and low strength. The post-treated impregnated epoxy resin can obtain a dense sample, and its bending strength and tensile strength were improved by 17.3% and 28%, respectively.
- For the GF/PF/EP three-phase composite materials containing 60 vol.%, 70 vol.%, and 80 vol.% of glass fibers, the tensile strength was 86.4, 92, and 96.2 MPa, respectively, and the flexural strength was 119, 129, and 137 MPa, respectively. Glass fiber is used as a reinforcing material, and as the GF content increases, the tensile strength and bending strength of the sample also increase.
- The electrical insulating properties of the composite material are enhanced with the increase of GF content. The breakdown voltage of the composite materials having a fiber content of 60 vol.%, 70 vol.%, and 80 vol.% was 29.6, 30.9, and 32.5 KV, and the DC volume resistivity was 19.978, 20.629 and 20.981 GΩ·m, respectively.

The results indicate that the mechanical and electrical insulation properties of the GF/PF/EP three-phase composite are enhanced with the increase of glass fiber content. This study lays the foundation for the fabrication of high-performance, complex, structural electrical insulation parts by SLS additive manufacturing technology.

## Figures and Tables

**Table 1:** The characteristics of the PF-2123 phenol formaldehyde resin.

Properties	Parameters
The average particle size (μm)	22.1
Softening temperature (°C)	98~115
Urotropine (%)	8~9
Density (g/cm^3^)	1.22
Material appearance	Light yellow to brown transparent solid

**Table 2:** Selective laser sintering (SLS) processing parameters for the GF/PF composites.

Laser Power (W)	Scan Velocity (mm/s)	Scan Spacing (mm)	Layer Thickness (mm)	Sintering Result
10	3000	0.2	0.1	Fragile
12	2500	0.3	0.1	Fragile
12	3000	0.2	0.1	Serious warped
12	3500	0.1	0.1	Serious warped
14	2500	0.1	0.1	Slightly warped
14	3000	0.2	0.1	Well formed
14	3500	0.3	0.1	Well formed
16	3000	0.2	0.1	Low precision

**Table 3:** Porosity of the SLS-sintered GF/PF green parts with different GF content.

GF Content/vol.%	60	70	80
Porosity/%	53.5	54.9	58.7

**Table 4:** Mechanical properties of the GF/PF/EP composites.

GF Content/vol.%	Tensile Strength/MPa	Bending Strength/MPa
60	86.4	119
70	92	129
80	96.2	137
